# Study of PEG/Biochar Cementitious Cold-Bonded Aggregate for Thermal Energy Storage

**DOI:** 10.3390/nano16080492

**Published:** 2026-04-21

**Authors:** Rongji Li, Chong Zhang, Yuechao Zhao, Changliang Wu, Guangbin Duan, Xiuzhi Zhang

**Affiliations:** School of Materials Science and Engineering, University of Jinan, Jinan 250022, China

**Keywords:** thermal energy storage, biochar, nanoscale pore structure, PEG, phase change aggregate, negative carbon emission

## Abstract

The incorporation of phase change materials in concrete is a practical strategy that holds great promise for enhancing the energy efficiency of buildings and reducing CO_2_ emissions. However, the direct contact between phase change materials and cement interferes with the cement hydration reaction, leading to a significant reduction in the mechanical strength of cementitious composites. To encapsulate polyethylene glycol and prevent leakage, this study developed a shape-stabilized phase change aggregate via the cold-bonding method and the vacuum impregnation method. The nanoscale pore structure of the aggregate was regulated by adjusting the biochar content to enhance the phase-change material loading capacity. The phase change aggregate was characterized by indicators including crushing strength and water absorption. Meanwhile, its microstructure, the correlations between nano-sized hydration products, chemical compatibility, and phase change properties were analyzed. The fabricated phase change aggregate has a crushing strength of over 5 MPa, latent heat of 42.84 J/g, and phase change temperature of 29.17 °C while also exhibiting good mechanical properties and thermal energy storage performance. The compressive strength of phase change concrete can meet the strength requirements for structural building material. Moreover, phase change aggregate contributed to reduced CO_2_ emissions during service, with favorable economic and low-carbon benefits over its service life, demonstrating good performance in both economic efficiency and CO_2_ emission reduction.

## 1. Introduction

In recent years, with the growth of the population and economy, global warming has become a severe challenge facing modern society. According to statistics from the International Energy Agency (IEA), buildings accounted for approximately 30% of the world’s energy consumption and more than half of the global electricity consumption in 2024 [1]. To achieve carbon neutrality by 2050, the construction industry must improve energy efficiency to reduce CO_2_ emissions. Given that heating and cooling constitute the primary energy consumption modes of buildings, enhancing the application of thermal energy storage technologies in buildings to reduce fossil energy consumption is a key approach to achieving building energy conservation and CO_2_ emission reduction [2].

Phase change material (PCM) is functional material that stores and regulates thermal energy by absorbing or releasing latent heat during phase transition processes. It exhibits significant application potential in the construction sector and has been identified as an effective strategy for reducing energy consumption [3]. However, the use of PCM in concrete is restricted, as PCM will inevitably degrade the mechanical properties of concrete and cause liquid leakage within the concrete matrix. Therefore, the encapsulation method of PCM is of critical importance.

Currently, common encapsulation methods are classified into two categories: microencapsulation [4] and macroencapsulation [5]. Microencapsulated PCM is composite functional material with a core–shell structure, which is prepared by confining PCM in tiny containers via microencapsulation technology to prevent its leakage. Some scholars have identified that the low load-bearing capacity of microencapsulated PCM [6] and the weak bonding interface and voids between microencapsulated PCM and the surrounding matrix [7] are the primary causes of the deterioration in concrete performance. In addition, the high preparation cost of microencapsulated PCM restricts its large-scale application.

To address the above issues, some researchers have proposed macroencapsulation, using porous aggregates with a certain mechanical strength to adsorb PCM and then encapsulating it to prepare phase change aggregate (PCA). Currently, commonly used inorganic porous aggregates mainly include expanded shale [8], expanded graphite [9], zeolite [10], and so on. However, the commonly used porous supporting materials suffer from poor strength, which leads to a decrease in the mechanical properties of heat-storing cementitious materials containing PCM. Thus, there is an urgent need to develop cost-effective, high-strength, and low-carbon PCA.

Artificial aggregate has been widely recognized by scholars worldwide due to its high strength, high porosity, and strong pore connectivity. At present, there are two main methods for manufacturing artificial aggregate: high-temperature sintering and cold-bonded granulation [11]. The most common artificial aggregate is sintered aggregate, which is produced under high-temperature conditions and generates high CO_2_ emission. In contrast, the cold-bonding process is a green production method that utilizes solid waste at room temperature with low CO_2_ emission. This type of artificial aggregate is referred to as cold-bonded aggregate (CBA), which has attracted increasing attention from numerous scholars in recent years [12,13,14].

Biochar, as a carbon-based material, has attracted extensive attention due to its excellent properties such as abundant nanoscale pore structure and high thermal conductivity [15]. However, the large-volume application of biochar in concrete is limited due to its inherent drawbacks such as high porosity and low load-bearing capacity. Recent studies have shown that artificial aggregate can avoid the performance degradation of mortar caused by biochar. For example, Zou et al. [16,17] prepared CBA using biochar and cement, realizing low-carbon production of building materials. This provides a new insight for the high-volume application of biochar in concrete. Biochar possesses a rich pore structure and a large specific surface area [18]. Biochar effectively sequesters atmospheric CO_2_ that was originally fixed by plants during photosynthesis. The carbon stored in biochar is highly stable and can offset the greenhouse gas emissions generated during its production process, rendering biochar a carbon-negative material [19]. Therefore, it is expected that the incorporation of biochar into CBA can increase the volume for PCM. Meanwhile, the stable carbon structure of biochar enables long-term carbon sequestration in cementitious materials, reducing the carbon footprint of building materials.

In contrast to lipid-soluble PCM, water-soluble PCM can be readily adsorbed into the pores of inorganic materials via polar interactions or van der Waals forces, thereby forming stable thermal energy storage structures [20]. This compatibility mechanism can be extended to PCA fabricated from inorganic porous aggregates such as ceramsite. As a representative water-soluble PCM, polyethylene glycol (PEG) possesses high latent heat, excellent stability, non-corrosiveness, flame retardancy, and biodegradability, demonstrating great potential for building applications [21].

This study prepared biochar-modified CBA by cold bonding, designed a shape-stabilized aggregate with cement as the coating, and loaded PEG to fabricate high-performance PCA. The effects of biochar content on the macroscopic physical properties and microscopic pore structure of the cold-bonded aggregates were also investigated. Polyethylene glycol (PEG) was selected as the PCM and adsorbed by the aggregates to fabricate PCAs. Differential Scanning Calorimetry (DSC), Fourier Transform Infrared Spectroscopy (FT-IR), and Thermogravimetry (TG) were employed to determine the influences of biochar content on the phase change parameters, chemical compatibility, and thermal stability of PCA. In addition, this study explored the correlation between the aggregate microstructure and its phase change performance and analyzed the influence mechanism of PCA on the physical properties of concrete. Finally, the economic assessment and CO_2_ emission of PCA were also investigated.

## 2. Materials and Methods

### 2.1. Materials

**Figure 1:**
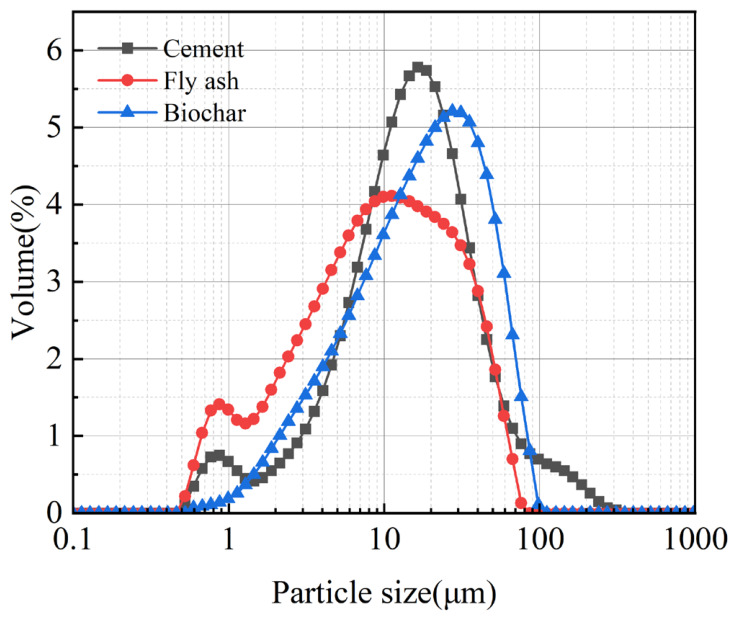
Particle size distribution of raw materials.

The aggregate material used in this study mainly include fly ash (FA) (Yixiang New Materials Co., Ltd., Henan Province, China) and ordinary Portland cement (OPC, P.O 4.25) (Bole New Materials Co., Ltd., Henan Province, China), with biochar employed to regulate the pore structure of the aggregate. Biochar(Lvzhiyuan Co., Ltd, Henan Province, China) was fabricated via pyrolysis of waste corn cobs at 500 °C for 2 h at a heating rate of 10 °C/min and a holding time of 60 min. The chemical compositions of OPC and fly ash were determined by Tiger S8 XRF (Bruker Co., Ltd., Germany) (Table 1). The elemental composition of biochar was measured using an EA3000 organic elemental analyzer (Eurovector Co., Ltd., Italy) (Table 2). Particle size distributions of OPC, biochar, and fly ash were analyzed by laser diffraction (Mastersizer 3000, Malvern, UK) (Figure 1). Polyethylene glycols with different molecular weights exhibit different phase change temperatures. Considering that the suitable temperature for the human body ranges from 20 to 30°C, polyethylene glycol 800 (industrial grade, purity > 98%, Biyang Industrial Co., Ltd., Shanghai, China), whose phase change temperature falls within this range, is selected as the phase change material. It has a melting point range of 20~30 °C, a melting enthalpy of 131.38 J/g, and a solidification enthalpy of 146.13 J/g.

In the concrete mixing process, the natural fine aggregate was river sand, with a particle size range of 0.8–5 mm, a bulk density of 1486 kg/m^3^, and a fineness modulus of 3.4, and the natural coarse aggregate was limestone, with a particle size range of 5–15 mm and a bulk density of 1493 kg/m^3^. To enhance the thermal insulation performance of concrete, the prepared PCA was used to replace natural coarse aggregate in concrete for the fabrication of phase change concrete.

### 2.2. Preparation of Cold-Bonded Aggregate (CBA) and Phase Change Aggregate (PCA)

Since fly ash exhibits no cementitious properties, replacing fly ash with biochar while maintaining a constant cement dosage can ensure that the ceramsite achieves relatively high strength. However, when biochar is used to replace cement, the mechanical properties of ceramsite inevitably deteriorate. To investigate the effect of different biochar content on aggregate properties, four groups of mix proportions were designed, as shown in Table 3.

The production process of CBA was conducted as follows: The weighed dry powders were first put into a mixer for premixing for 2 min, followed by adding an appropriate amount of water and mixing for another 3 min. This avoided spilling of raw materials during the granulation process and allowed for more accurate control of the water-to-binder ratio. Subsequently, the mixed raw materials were fed into the granulator with a disc diameter of 500 mm and a height of 200 mm, and a proper amount of water was sprayed onto the mixture to promote particle nucleation and agglomeration. Based on previous research [16,22], the operating parameters of the granulator were determined as follows: rotating speed of 30 r/min, inclination angle of 45°, and granulation duration of 20 min. Afterwards, dense and hardened fresh spherical particles were obtained, which were then cured in a steam curing box at 60°C for 3 days.

**Figure 2:**
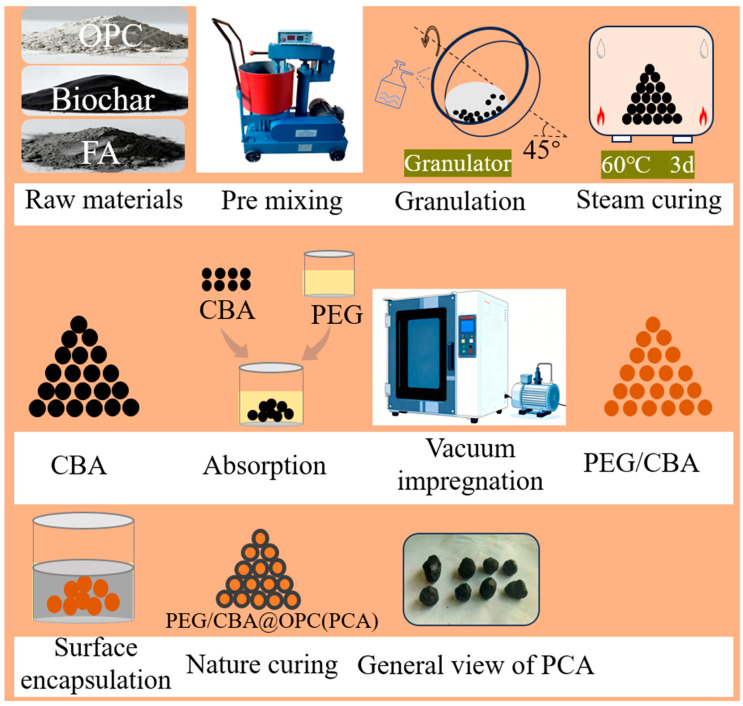
Schematic illustration of preparing phase change aggregate.

The particles (CBA) and PEG mixed in a beaker were placed in a vacuum drying oven and heated at −0.08 MPa and 60 °C for 2 h. The impregnated particles (PEG/CBA) were coated with OPC slurry at a water/binder ratio of 0.5 to avoid PEG leakage. Finally, the coated particles were cured at room temperature to obtain PEG/CBA@OPC. The production process flow of PEG/CBA@OPC is illustrated in Figure 2.

### 2.3. Mix Proportion of Phase Change Concrete

As shown in Table 4, the developed PEG/CBA@OPC (PCA) with biochar content of 50% was used to replace natural coarse aggregate by volume for concrete production. For the phase-change concrete reference group PCAC0, only natural aggregate was adopted, with a water/binder ratio of 0.39 for the concrete. Since lightweight aggregate generally has a much higher water content and water absorption rate than natural aggregate, the natural aggregate and phase change ceramsite were soaked for 24 h and then air-dried to achieve a surface-dry saturated state. First, the aggregates and one-third of the water were added to the mixer and stirred for 60 s to fully moisten the surfaces of the aggregates. Then, 50% of the cement was added and mixed for 1 min to ensure that the aggregate surfaces were uniformly coated with a cement paste film. Subsequently, the remaining cement and water were added to the mixer and mixing continued for 2 min. After mixing, the mixture was placed into molds and compacted manually in layers to avoid internal voids caused by static slurry settlement. The specimens were covered with plastic film, demolded after 24 h, and then transferred to a standard curing room for curing.

### 2.4. Test Methods

#### 2.4.1. Aggregate Properties

After curing, the aggregates were tested for their loose bulk density, water absorption, and crushing strength. The test trials were conducted in accordance with the Chinese national standard (GB-T 17431.2-2010). The loose bulk density (ρ, kg/m^3^), 1 h water absorption (ω, %), and crushing strength ($f_{a}$, MPa) were calculated based on Equations (1)–(3):

(1) $$f_{a}=\frac{p_{1}+p_{2}}{F}$$

(2) $$\rho =\frac{m}{V}$$

(3) $$\omega =\frac{m_{1}-m}{m}$$

where $f_{a}$ is the crushing strength of aggregates (MPa), $p_{1}$ is the pressure at a depth of 20 mm (N), $p_{2}$ is the mass of the piston (N), and F is the area of the piston (mm^2^); ρ is the bulk density (kg/m^3^), m is the mass of the sample (kg), and V is the volume of the measuring cylinder (L); and ω is water absorption (%), $m_{1}$ is the mass of the soaked sample (g), and m is the mass of the dried sample (g). Three parallel tests were carried out for each group of specimens, and the average value was taken as the test result.

#### 2.4.2. Concrete Properties

The apparent density was tested in accordance with GB/T 50080-2016. The volumetric cylinder method was used to determine the apparent density of fresh concrete. The concrete mixture was placed into the measuring cylinder in three layers; each layer was tamped 25 times with a tamping rod. After filling and leveling the surface, the total mass of the cylinder and sample was weighed, and the apparent density was calculated by Equation (4):

(4) $$\rho =\frac{m_{2}-m_{1}}{V}$$

where ρ is the apparent density of concrete (kg/m^3^); $m_{1}$ is the mass of the measuring cylinder (kg); $m_{2}$ is the total mass of the measuring cylinder and concrete (kg); and V is the volume of the measuring cylinder (L).

Each group was tested in duplicate, and the average value was taken as the test result.

The mechanical properties of concrete specimens cured to the specified age were tested. The uniaxial compressive strength of concrete was measured according to the GB/T 50081-2019 Standard for Test Methods of Physical and Mechanical Properties of Concrete. Before testing, the oil pump was started. The concrete specimen was placed at the center of the lower platen of the compression machine, and then the machine was controlled to apply a constant load at a rate of 3–5 kN/s until the specimen failed. The loading was stopped, and the maximum compressive stress at failure was recorded. The compressive strength of concrete was calculated by Equation (5):

(5) $$f=\frac{F}{A}$$

where f is the uniaxial compressive strength of the cubic concrete specimen (MPa); F is the maximum failure load of the cubic concrete specimen (N); and A is the bearing area of the cubic concrete specimen (mm^2^).

Cubic specimens of 100 mm × 100 mm × 100 mm were used, with three specimens per group. The arithmetic mean was taken as the representative value after eliminating outliers. All test results were multiplied by a reduction factor of 0.85.

#### 2.4.3. FT-IR Analysis

A Nicolet 380 FT-IR spectrometer (Thermo Fisher Scientific, Waltham, MA, USA) was employed to characterize the chemical compatibility of phase change ceramsite. Before testing, ceramsite samples were soaked in isopropanol for 1 d to stop hydration and then dried in a vacuum drying oven for 1 d. The ceramsite was crushed, ground into powder, and sieved through a 200-mesh sieve. The powder was mixed with KBr at a mass ratio of 1:100 and ground uniformly. The mixture was pressed into pellets using a hydraulic press at 5 MPa. Finally, the pellet samples were placed into the sample chamber of the spectrometer to collect FT-IR spectra. The scanning wavenumber range was 400–4000 cm^−1^, and the spectral resolution of FT-IR was 0.5 cm^−1^. The test data were processed to plot FT-IR curves, which were used to analyze the changes in characteristic peaks of the phase change material. Three parallel tests were carried out for each group of specimens, and the average value was taken as the test result.

#### 2.4.4. TG Analysis

Ceramsite samples were soaked in isopropanol for 1 d to terminate hydration and then dried in a vacuum drying oven for 1 d. The ceramsite was crushed, ground into powder, and sieved through a 200-mesh sieve. Thermogravimetric analysis measurements were performed on a STA200 (HITACHI, Tokyo, Japan) simultaneous thermal analyzer with a heating rate of 20 °C/min from room temperature to 800°C under N_2_ atmosphere. Three parallel tests were carried out for each group of specimens, and the average value was taken as the test result.

#### 2.4.5. SEM Analysis

The microstructure and morphology of the samples were characterized using a EVOLS15 scanning electron microscope (SEM) (Carl Zeiss Optics (China) Co., Ltd., Guangzhou, Guangdong Province, China). The ceramsite samples were crushed into uniform slices with moderate thicknesses and smooth surfaces. Hydration of the slices was terminated with isopropanol, followed by drying in a vacuum drying oven for 1 d to completely remove internal moisture and residual isopropanol. The prepared ceramsite slices were evenly fixed on the SEM stage with conductive adhesive and sputter-coated with gold uniformly within 180 s. Microstructural observations were performed at an accelerating voltage of 5 kV.

#### 2.4.6. LF-NMR Analysis

In this study, low-field nuclear magnetic resonance (LF-NMR) was used to characterize and analyze the pore structures of cold-bonded aggregates. The final results of each test were determined through repeated experiments.

The paste of cold-bonded aggregate was cast into cylindrical specimens with a diameter of 20 mm and a height of 20 mm, followed by vacuum saturation in deionized water for 72 h. The T_2_ relaxation time of the specimens was then measured using a Niumag low-field nuclear magnetic resonance analyzer with the following parameters: 12 MHz pulse frequency, 0.1 ms echo time, and 2000 echoes. Finally, the pore size distribution of the specimens was calculated using Equation (6):

(6) $$\frac{1}{T_{2}}\approx \frac{1}{T_{2\;sur}}=\rho_{2}\times \left(\frac{S}{V}\right)=\rho_{2}\times \frac{2}{r}$$

where $T_{2}$ represents the relaxation time of water in the pores; $T_{2\;sur}$ is the surface relaxation time; $\rho_{2}$ is the surface relaxivity, taken as 5 μm/s [23]; $\frac{S}{V}$ is the ratio of surface area to pore volume; and r is the radius of the pore.

#### 2.4.7. DSC Analysis

The phase change enthalpy and phase change temperature of PEG and PCA were measured using a DSC3 differential scanning calorimeter (Mettler toledo, Switzerland). After crushing, samples were randomly taken from the core of the ceramsite, ground into powder, and sieved through a 200-mesh sieve. The test temperature ranged from 0 to 60 °C, with both heating and cooling rates set at 5 °C/min. Three parallel tests were carried out for each group of specimens, and the average value was taken as the test result. Three parallel tests were carried out for each group of specimens, and the average value was taken as the test result.

## 3. Results and Discussion

### 3.1. Effect of Biochar on Physical Properties of PCA

**Figure 3:**
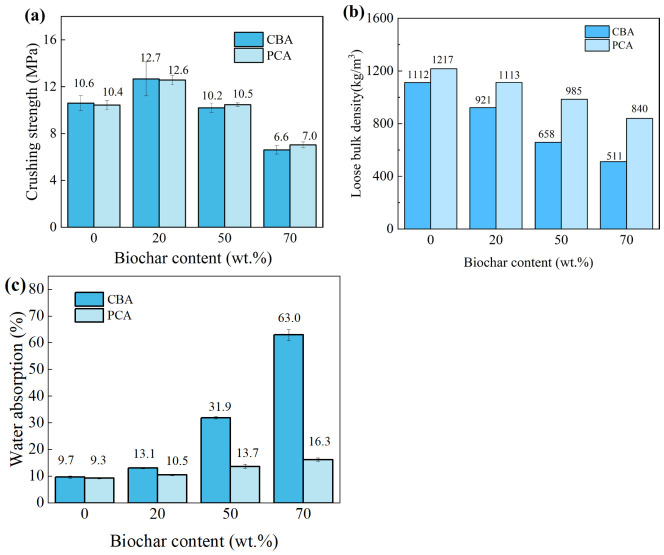
(**a**) Crushing strength, (**b**) loose bulk density, and (**c**) water absorption of CBAs and PCAs with different biochar contents.

To evaluate the effect of biochar content on the performance of phase change aggregates, aggregates with different biochar dosages (0%, 20%, 50%, and 70%) were selected for impregnation and coating experiments. Figure 3a, b, and c show crushing strength, loose bulk density, and water absorption of the four groups of aggregate before and after impregnation and coating, respectively.

It can be seen in Figure 3a that the crushing strength of the aggregate first increased and then decreased with the increase in biochar content. CBA20 exhibits the highest strength, with a crushing strength of approximately 12.7 MPa. This strength enhancement is attributed to the porous nature of biochar, as it provides additional water for hydration inside the aggregate. Biochar absorbs and stores extra water during the initial mixing stage and then releases the water in the later cement hydration stage to promote the hydration reaction [24]. In addition, the large specific surface area of biochar provides extra surfaces for hydration nucleation, thereby accelerating the hydration reaction and leading to faster hardening and densification of the cement matrix. Steam curing enhances early-age strength development and promotes the formation of cement hydration products, which, in turn, enhances the densification of the internal structure and reduces porosity, consequently accelerating the strength development of the cementitious system. Nevertheless, when the biochar content exceeds 20%, the compressive strength of CBA decreases significantly. This phenomenon is mainly attributed to the intrinsic porous structure and weak load-bearing capacity of biochar itself. The strength of aggregate with low biochar content decreases after coating, while the coating layer improves the crushing strength of aggregate with high biochar content, which can be attributed to two reasons: the weak interfacial transition zone and cement hydration reaction. However, the impregnation and coating of aggregate have a minimal effect on crushing strength, proving that the performance of PCA is stable.

For concrete aggregate, apart from crushing strength, loose bulk density is a core parameter characterizing the particle packing state, which is directly related to the volume stability of concrete. It can be seen in Figure 3b that the loose bulk density of cold-bonded aggregate is negatively correlated with biochar content. When the biochar content increases from 0 to 70%, the loose bulk density of CBA decreases by about 54% (from 1114 kg/m^3^ to 511 kg/m^3^). The reason for this reduction is that the lightweight and porous structure of biochar itself causes the aggregate density to decrease with the increase in biochar content. These results indicate that the incorporation of biochar is beneficial to the production of lightweight artificial aggregate. In addition, the impregnation of PEG and OPC slurry coating have a certain impact on sample density. The loose bulk densities of the four groups of PCAs correspond to the water adsorption rate of aggregate, which proves that the aggregate performance is uniform.

Water absorption is a core indicator characterizing the pore structure and interfacial compatibility of aggregate. It can be seen in Figure 3c that the water absorption of CBA increases rapidly with the increase in biochar content, which is mainly attributed to the loose pore structure of biochar itself. In addition, high-content biochar reduces the hydration reaction of cementitious materials and the formation of crystalline products, thereby increasing the aggregate porosity. Notably, after impregnation with PEG and coating with OPC slurry, the water absorption of the four groups of PCAs is significantly reduced. This may be because the outer OPC slurry coating has low water absorption and PEG fills the aggregate pores to limit the permeability of internal water, resulting in a significant reduction in water absorption.

### 3.2. Effect of Biochar on Microstructure of CBA

**Figure 4:**
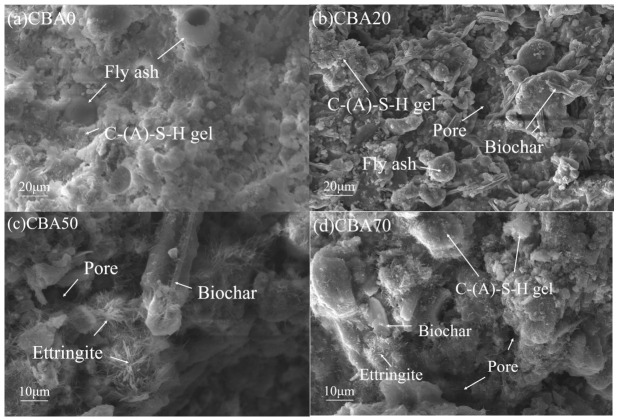
SEM images of CBAs with different biochar contents.

The effects of different biochar contents on the pore structure of CBA were investigated via SEM and LF-NMR. Figure 4a–d present the SEM micrographs of CBAs with different biochar contents. It can be observed in the SEM images that the matrix of CBA is composed of nano-sized C-S-H gel (amorphous flocculent structure) and spherical fly ash particles, while biochar exhibits a hollow long rod-like structure and is uniformly dispersed in the CBA. A large amount of C-S-H gel is present around biochar, which is likely because water released from biochar promotes the formation of cement hydration products, thereby contributing to a certain improvement in the strength of CBA. There are also several pores in the CBA, which is related to the granulation process during its preparation. A fully dense structure is difficult to form, which results in the reduction of the aggregate mechanical strength at high biochar contents. The incorporation of biochar and the granulation process construct a hierarchical pore structure dominated by nanopores for CBA, providing sufficient storage space for PEG at the nanoscale, and the uniform dispersion of biochar ensures the homogeneity of the nanoscale pore structure of aggregates.

**Figure 5:**
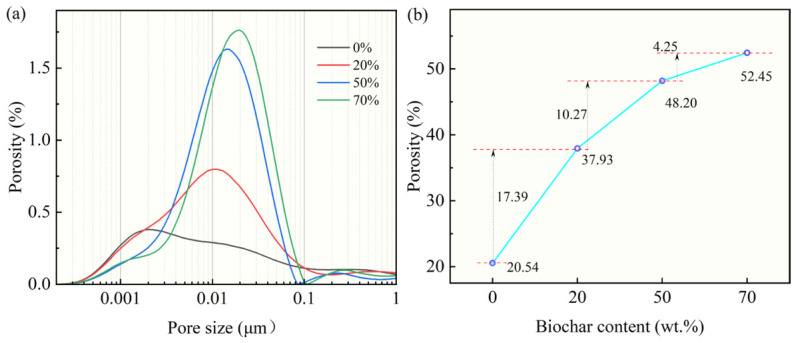
Pore size distribution and total porosity of CBAs with different biochar content. (**a**) Pore size distribution; (**b**) Total porosity.

Figure 5a,b show the pore size distribution and total porosity of CBA, respectively. The pore sizes of the CBA were mainly distributed in the nanoscale range of 1–100 nm. The addition of biochar significantly altered the nanopore distribution of the CBA. The pore structure can be divided into four categories: harmless pores (<20 nm), slightly harmful pores (20–50 nm), harmful pores (50–200 nm), and seriously harmful pores (>200 nm) [25]. The total porosity of the aggregate increased with the rise in biochar content, especially for pores in the range of 1–100 nm, which leads to a decrease in the mechanical strength of the aggregate. Overall, the incorporation of biochar introduced abundant capillary pores into the CBA, thereby providing adequate space for the subsequent PEG impregnation.

### 3.3. Effect of Biochar on Chemical Compatibility of PCA

**Figure 6:**
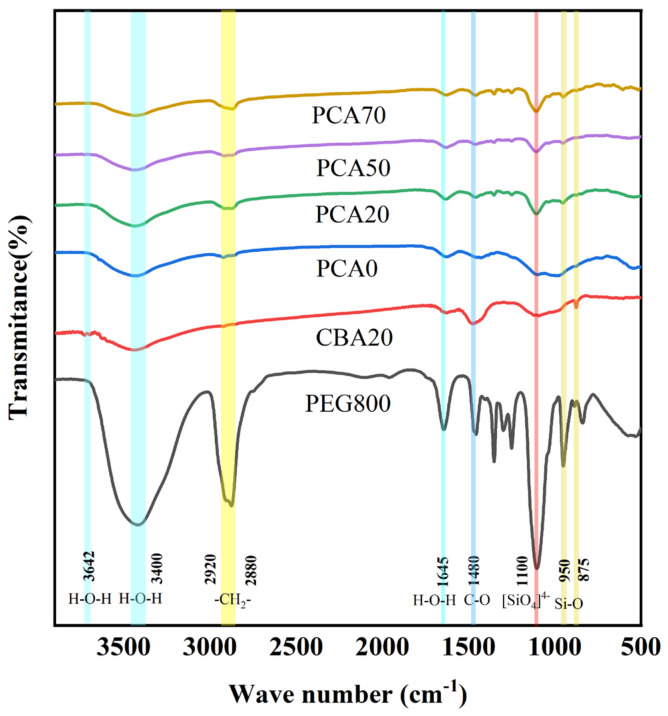
FT-IR curves of PEG, CBA, and PCA.

FT-IR was employed in this study to characterize the chemical compatibility of PCA components. As illustrated in Figure 6, the FT-IR of CBA20 exhibited characteristic peaks in the range of 875 cm^−1^ to 950 cm^−1^, which were assigned to the stretching vibrations of Si-O functional groups in C-S-H gel. The peak detected at 1100 cm^−1^ corresponded to the vibrations of [SiO_4_]^4−^ tetrahedra associated with ettringite (Aft), while the peak at 1480 cm^−1^ was attributed to the stretching vibrations of C-O functional groups in calcite. The absorption bands at 2880 cm^−1^ and 2920 cm^−1^ were, respectively, assigned to the symmetric and asymmetric stretching vibrations of -CH_2_- (methylene) groups in biochar [26]. In addition, characteristic bending peaks of H-O-H in water were observed at 3642 cm^−1^, 3400 cm^−1^, and 1645 cm^−1^ [27], with the peak at 3642 cm^−1^ corresponding to the O-H stretching vibrations in Ca (OH)_2_. These results further confirm that the main hydration products of CBA are ettringite and C-S-H gel.

Strong absorption peaks at 2880 cm^−1^ and 2920 cm^−1^ were also observed, corresponding to the symmetric and asymmetric stretching vibrations of methylene (-CH_2_-) groups in PEG, and the intense peak at 1100 cm^−1^ was associated with the asymmetric stretching vibrations of ether linkages (C-O-C) in PEG. The characteristic peaks of PEG in PCA0 are not obvious, indicating that the adsorption amount of PEG in PCA0 is limited and the aggregate structure is relatively dense. In contrast, distinct PEG characteristic peaks were detected in PCA20, PCA50, and PCA70, demonstrating that the incorporation of biochar effectively enhanced the PEG loading capacity of the aggregate.

Besides the above-mentioned variations, all PCA spectra displayed the characteristic peaks of both PEG and CBA without the emergence of any new absorption bands. It can therefore be concluded that no chemical reaction occurred between CBA and PEG, and the prepared PCAs possessed good chemical compatibility.

### 3.4. Effect of Biochar on Thermal Stability of PCA

**Figure 7:**
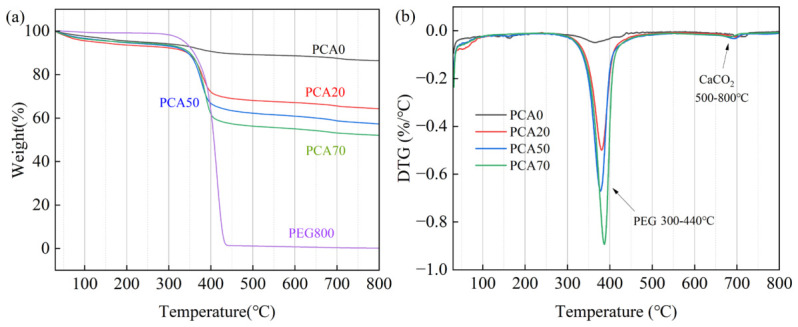
(**a**) TG curves of PEG and PCA; (**b**) DTG curves of PCA.

Figure 7a,b show the TG and DTG curves of PEG and PCA. The distinct weight loss peak observed between 300 °C and 440 °C corresponds to the decomposition of PEG, which is attributed to the cleavage of PEG molecular chains under high-temperature conditions. It could also be observed from the TG curves that the residual mass of PCA decreased sequentially, indicating that an increase in biochar content could enhance the adsorption capacity of PCA for phase change materials. The broad weight loss region between 50 °C and 380 °C was mainly caused by the dehydration of calcium silicate hydrate (C-S-H) in cement. The main water loss of C-S-H took place between 50 °C and 160 °C, and the dehydration process continued up to 600 °C [28]. In addition, the weight loss within this range was associated with the evaporation of free water from the pores and gel layer of the matrix [29]. The weight loss observed between 500 °C and 800 °C was ascribed to the decomposition of CaCO_3_, which produced CaO and CO_2_, with the escape of CO_2_ leading to the reduction in weight [30]. It is worth noting that the surface temperature of the exterior wall of buildings generally does not exceed 70 °C–80 °C [31]. Therefore, it can be concluded that the PCAs prepared in this study exhibit good thermal stability for applications in construction.

### 3.5. Effect of Biochar on Phase Change Properties and Form-Stable Ability of PCA

**Figure 8:**
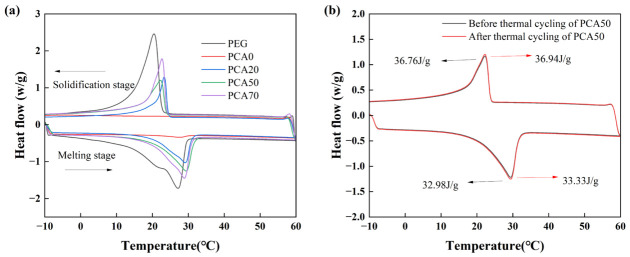
(**a**) DSC curves of PEG and PCA; (**b**) DSC curves of PCA50 before and after 100 thermal cycles.

The DSC curves in Figure 8 show the peak temperature of phase change enthalpy of PEG and PCA during melting or solidification processes. The latent heat values and peak phase change temperatures of PEG, PCA0, PCA20, PCA50, and PCA70 during melting and solidification processes are presented in Table 5. Compared with PEG, PCA0 exhibited a very low melting enthalpy, which could be attributed to the dense structure formed by cement hydration that resulted in the lack of thermal storage capacity of CBA. Notably, the melting enthalpy of PCA20 is considerably higher than that of PCA0, and the melting enthalpy of PCA increases with the rise in biochar content. The enhancement in thermal storage performance can be attributed to the pore-forming effect of biochar, which facilitates an increase in the loading capacity of PEG. In addition, it is found from the DSC curves in Figure 8a that the peak phase change temperature of PCA in the melting stage is significantly higher than that of PEG, which is inconsistent with the result reported in reference [32] that the phase change temperature of PCA is similar to that of pure PEG. Zhang et al. [33] proposed that this is attributed to the low thermal conductivity of the sintered aggregate skeleton and the small amount of air in the internal pores of sintered aggregate, which hinders the heat transfer efficiency from the external environment to PEG. Moreover, pure PEG undergoes volume expansion during melting; thus, the PEG confined in the internal pores of PCA leads to an increase in pore air pressure under heating conditions, resulting in the elevated phase change temperature of PEG in PCA [34]. In summary, the difference in phase change temperature between PCA and PEG can be explained by the combined effect of these two factors. Figure 8b presents the DSC curves of PCA50 before and after 200 thermal cycles. The DSC curves indicate that there is almost no difference in the phase change performance of the aggregate before and after thermal cycling, which demonstrates that the prepared PCAs possess good thermal cycling reliability. Overall, the developed PCA exhibits good thermal reliability and can be regarded as a promising candidate for thermal energy storage media in building envelopes.

**Figure 9:**
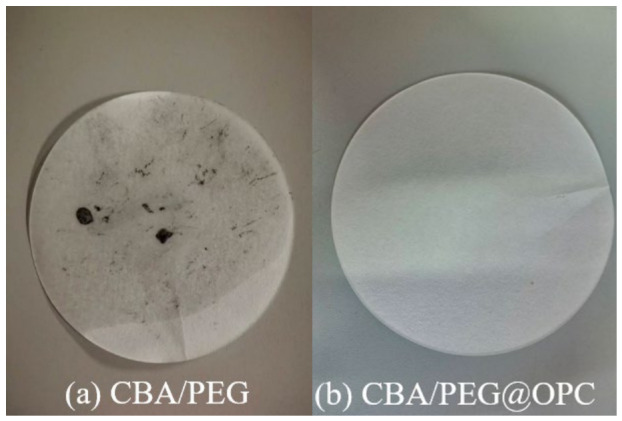
(**a**) CBA/PEG at 60 °C for 1 h; (**b**) CBA/PEG@OPC.

To evaluate the form-stable ability CBA/PEG of and CBA/PEG@OPC, samples were transferred to a chamber of 60 °C for 1 h. Significant leakage of CBA/PEG was observed (Figure 9a). It turned out that CBA/PEG@OPC exhibited no discernible traces of leakage (Figure 9b), confirming its superior form-stable ability.

### 3.6. Effect of PCA on Mechanical Properties of Concrete

Table 6 presents the apparent density and 28 d compressive strength of concrete with a PCA50 replacement ratio of 0%, 20%, 40%, 60%, and 80%, respectively. With the increase in PCA50 replacement ratio, the apparent density and 28 d compressive strength of concrete decrease continuously from 2210 kg/m^3^ and 51.2 MPa for PCC0 to 1942 kg/m^3^ and 21.8 MPa for PCC80, respectively. This reduction is attributed to the lower strength of artificial aggregate compared with natural aggregate, thus exerting a negative effect on the overall compressive strength of phase change concrete. Similar results in other studies [13,35] have also reported this phenomenon. The developed PCAC80 concrete containing 80% PCA50 is lightweight concrete, with a density lower than 1950 kg/m^3^ and a 28 d compressive strength of 21.8 MPa.

Notably, these analogous experimental results indicate that the reduction rates of 28 d compressive strength reach up to 55.02% and 51.83% when PCA prepared with expanded vermiculite and expanded perlite as carrier materials is added at 50 vol% in phase change concrete, which are higher than that of the PCA prepared in this work (49.8%). The difference in the reduction rate is due to the superior mechanical support provided by aggregate, a property that other porous aggregate such as expanded vermiculite and expanded perlite does not possess. In terms of 28 d compressive strength, the compressive strength of phase change concrete exceeds 30 MPa at a 40% mass replacement ratio of PCA. Therefore, the incorporation of 40% PCA in concrete wall construction can maximize the temperature-regulating capacity while meeting the required compressive strength. Table 7 shows the comparison results of compressive strength for different phase change concretes.

### 3.7. Economic Evaluation and CO_2_ Emission

**Figure 10:**
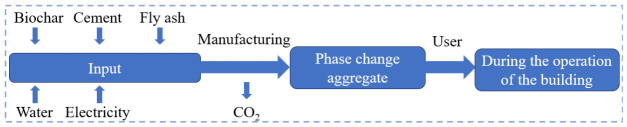
LCA results for phase change aggregate: system boundary of the phase change aggregate used in passive solar buildings.

Figure 10 illustrates the system boundary of phase change aggregates in the Life-Cycle Assessment (LCA) model. In the PCA system, biochar, serving as a pore-forming agent, enhances the load-bearing capacity of PCAs, which, in turn, improves the thermal energy storage performance of artificial aggregates while enabling carbon sequestration. To assess the sustainability of the developed PCAs, a “cradle-to-grave” LCA model was employed. The system boundary encompasses three key stages: raw material procurement, product manufacturing, and operational use.

The carbon emissions per unit of phase change ceramsite is calculated by Equation (7):

(7) $${SUM}_{ce}=\sum_{i=1}^{n}Cef_{mi}*M_{i}+\sum_{i=1}^{n}Cef_{ti}*T_{i}*M_{i}+Cef_{p}*E_{p}$$

where $Cef_{mi}$ is the carbon emission factor of materials, including cement, biochar, and water, as shown in Table 8; $M_{i}$ is the material consumption required to produce one functional unit of ceramsite (Table 3); $Cef_{ti}$ is the carbon emission factor for highway transportation (Table 8); and $T_{i}$ is the transportation distance of materials (Table 9). In this study, the energy consumption for granulation is 4.1 kW, and the CO_2_ emission factor of electricity is 0.58 kg/(kW·h). The carbon emission factor of the granulation process ($Cef_{p}$) is calculated as 2.378 kg CO_2_ e/h. Given that the granulation time is set to 20 min and the output per batch is 40 kg, the granulation efficiency ($E_{p}$) is calculated as 0.12 t/h.

During the operation of the building, PCAs can be integrated into solar buildings since their phase change temperature falls within the residential thermal comfort range (20 °C∼28 °C). PCA can absorb and store excess solar radiation during the daytime when the ambient temperature exceeds the melting point. At night, when the temperature drops below the melting point, PCA can release the stored thermal energy into the indoor environment. This process significantly mitigates indoor temperature fluctuations and reduces reliance on active heating systems, thereby lowering energy consumption and carbon emission while improving thermal comfort.

In this work, Jinan City was selected as a representative city to evaluate the applicability of PCA in passive solar buildings. Through optimized design, a complete charge–discharge cycle of PCA can be achieved within a day and night cycle. Therefore, the theoretical daily energy savings are equivalent to its melting enthalpy. The annual energy savings of PCA in passive solar buildings can be expressed by Equation (8) [41]:

(8) $$E_{s}=\Delta H_{m}*m*D_{sum},$$

where $E_{s}$ is the annual energy saving of PCA in passive solar buildings, $\Delta H_{m}$ is the melting enthalpy of PCA, m is the mass of PCA, and $D_{sum}$ is the average annual number of sunny days in Jinan (i.e., 242 d).

**Figure 11:**
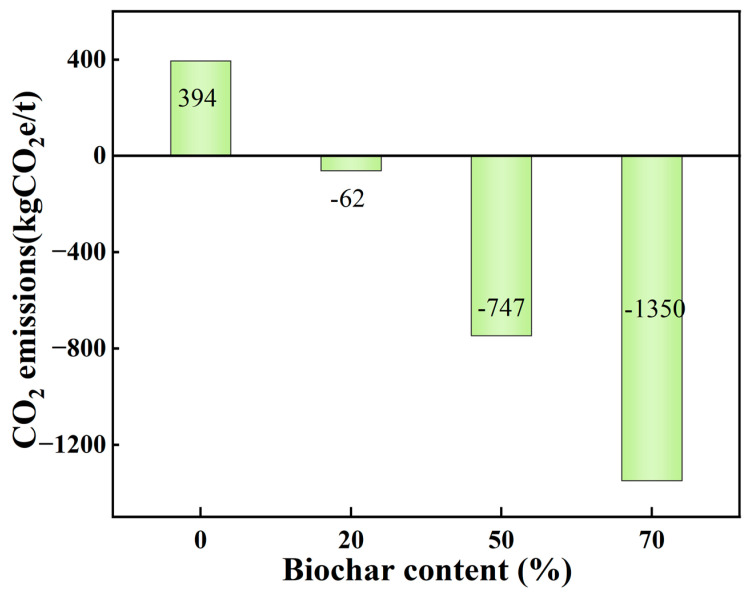
Impact of biochar content on CO_2_ emissions from 1 t CBA.

The impact of replacing cement and fly ash with biochar on CO_2_ emissions from biochar lightweight aggregates is shown in Figure 11.

Producing 1 t of lightweight biochar aggregate using 50% cement and 50% biochar results in CO_2_ emissions of −747.6 kg, including −782.5 kg from materials, 15.1 kg from transportation, and 19.8 kg from preparation. This confirms the environmental benefits of low-temperature granulation technology in lightweight aggregate production and demonstrates that CO_2_ emissions from lightweight biochar aggregate can be reduced at the material level.

It has been demonstrated that the incorporation of 1 kg of PCA50 into a passive solar building yields annual energy savings of 5.33 kW·h and a reduction of 2.24 kg in CO_2_ emissions. Based on the urban electricity price of approximately 0.60 CNY/(kW·h) in Jinan, the annual energy-saving benefit is calculated to be 3.198 CNY/km. Further analysis shows that the payback period is about 0.75 year, confirming the full financial viability of this technology. Table 10 presents a comparison of the raw material cost estimates based on the mix proportions used in this study, where all unit prices of raw materials are in accordance with the current market prices. Table 11 summarizes the aforementioned evaluation indicators.

The PCA developed in this study shows great potential for application in energy-efficient buildings. Furthermore, the concrete produced using PCA achieves high-volume biochar utilization while maintaining satisfactory structural performance. This not only enables carbon sequestration during concrete production but also reduces energy consumption during the building operational phase due to its lightweight nature and good thermal performance, thereby contributing to the sustainable development of the construction industry.

## 4. Conclusions

In this work, a novel shape-stabilized PEG/biochar cementitious PCA was developed via the cold-bonding method and vacuum impregnation method, aiming to improve the thermal performance of buildings and reduce CO_2_ emission. This study draws the following conclusions:

(1) The crushing strength of the developed PCA exceeds 5 MPa with a bulk density below 1200 kg/m^3^, falling into the category of lightweight aggregate.
(2) The nanopore-dominated pore structure of the aggregate was improved by adding biochar, thereby increasing the loading capacity of phase change materials. Meanwhile, a cement paste surface coating was introduced to ensure the shape stability of the PCA.
(3) The latent heat and peak phase change temperature of the PCA during melting and solidification stages are 42.84 J/g and 29.17°C and 40.54 J/g and 22.33 °C, respectively, which are suitable for residential thermal comfort. The PCA exhibits good chemical compatibility and thermal stability, with no interaction between components and no decomposition at temperature below 200 °C.
(4) With the increase in the dosage of PCA, the compressive strength of phase change concrete shows a decreasing trend. The 28 d compressive strength of PCC40 phase change concrete can reach 35.9 MPa, which meets the strength requirements for structural concrete.
(5) Incorporating 1 kg phase change aggregate into passive solar buildings yields significant environmental and economic benefits: 5.33 kW·h annual energy savings, 2.24 kg less CO_2_ emission, and CNY 3.198 in annual cost reductions, all with a dynamic payback period of merely 0.75 year.

## Figures and Tables

**Table 1:** Chemical compositions of raw materials.

	CaO	SiO_2_	Al_2_O_3_	Fe_2_O_3_	MgO	SO_3_	Na_2_O	K_2_O	Others
OPC	55.57	19.53	6.85	2.70	3.09	3.16	0.42	0.65	8.03
FA	1.80	50.90	39.66	2.19	0.70	1.27	0.87	1.08	1.53

**Table 2:** Elemental composition of biochar.

C	H	N	S	O
47.89	5.89	0.42	0.01	43.4

**Table 3:** Mix proportion of aggregate.

Mix	OPC (wt.%)	Biochar (wt.%)	FA (wt.%)	Water/Solid Ratio
CBA0	50	0	50	0.28
CBA20	50	20	30	0.35
CBA50	50	50	0	0.65
CBA70	30	70	0	0.85

**Table 4:** Mix proportion of concrete (kg/m^3^).

Mix	OPC	Water	Natural Fine Aggregate	Natural Coarse Aggregate	PCA50
PCAC0	444	173	634	938	0
PCAC20	444	173	634	750	126
PCAC40	444	173	634	563	252
PCAC60	444	173	634	375	378
PCAC80	444	173	634	188	496

**Table 5:** The latent heat and peak phase change temperature of PEG and PCAs with different biochar contents during melting and solidification stages.

Sample	Melting Stage		Solidification Stage	
	Latent Heat (J/g)	Temperature (°C)	Latent Heat (J/g)	Temperature (°C)
PEG	131.38	27.17	146.13	20.45
PCA0	0	-	0	-
PCA20	30.29	29.33	25.81	23
PCA50	36.94	29.67	33.33	22
PCA70	42.84	29.17	40.54	22.33

**Table 6:** Physical properties of phase change concrete.

Mix	Apparent Density (kg/m^3^)	28 d Compressive Strength (MPa)	Strength Reduction Rate
PCAC0	2210	51.2	0%
PCAC20	2115	43.0	16.2%
PCAC40	2072	35.9	29.9%
PCAC60	2015	25.7	49.8%
PCAC80	1942	21.8	57.4%

**Table 7:** Comparison of compressive strengths of different phase change concretes.

Phase Change Aggregate Composition	Phase Change Aggregate Fraction	28 d Compressive Strength (MPa)	Strength Reduction Rate	References
PEG/cold bonded aggregate	40%	35.9	29.9%	This work
	60%	25.7	49.8%	This work
Paraffin/non-sintered aggregate	50%	30.65	31.9%	[36]
PEG/expanded perlite	50%	19.64	51.83%	[35]
PEG/expanded vermiculite	50%	18.32	55.02%	[35]
PEG/ceramic aggregate	40%	33	38.23%	[37]
Paraffin/ceramic aggregate	40%	28	52.78%	[38]

**Table 8:** Carbon emission factors.

Materials and Processes	CO_2_ Emission Factor	Reference
Cement	735 kg CO_2_ e/t	GB/T 51336–2019
Fly ash	8 kg CO_2_ e/t	[39]
Biochar	−2300 kg CO_2_ e/t	[40]
Water	0.168 kg CO_2_ e/t	GB/T 51336–2019
Transportation (highway)	0.078 kg CO_2_ e/(t·km)	GB/T 51336–2019 (30 t truck)
Manufacturing	2.378 kg CO_2_ e/h	Ministry of Ecology and Environment of China

**Table 9:** Raw materials, transportation methods, and transportation distances.

Materials	Transportation Method and Distance
Cement	30 t truck (38 km)
Fly ash	30 t truck (40 km)
Biochar	30 t truck (348 km)

**Table 10:** Cost of raw materials.

Raw Materials	Usage Cost (CNY/kg)	Company
Biochar	2.45	Lvzhiyuan Co., Ltd., Henan Province, China
P.O 42.5 cement	0.28	Bole New Materials Co., Ltd., Henan Province, China
PEG800	7.60	Biyang Industrial Co., Ltd., Shanghai, China Compaina

**Table 11:** Economic evaluation and CO_2_ emission of PCA50 (kg^−1^).

Evaluation Contents	Evaluating Indicators
Cost recovery period	0.75 year
Annual energy savings	5.33 kW·h
Annual energy cost savings	3.198 CNY
Annual carbon dioxide emission reduction	2.24 kg

## Data Availability

No data were used for the research described in the article.
